# Repeated Annual Influenza Vaccination in Older Adults Induces Comparable Seroprotection Despite Reduced Antibody Fold Rise: A 6-Month Prospective Cohort Study in China

**DOI:** 10.3390/vaccines14040338

**Published:** 2026-04-11

**Authors:** Rong Wang, Tianchi Yang, Pingping Li, Baojun Li, Weibo Dong, Jingjing Wang, Lixia Ye

**Affiliations:** 1 Department of Virus Research, Ningbo Municipal Center for Disease Control and Prevention, Ningbo 315010, China; wr202502@126.com; 2 Department of Personnel and Education, Ningbo Municipal Center for Disease Control and Prevention, Ningbo 315010, China; cn-yangtc@outlook.com; 3 Department of Immunization Program, Jiangbei District Center for Disease Prevention and Control of Ningbo City, Ningbo 315021, China; lipingping0218@163.com; 4 Department of Infectious Disease Control, Haishu District Center for Disease Prevention and Control of Ningbo City, Ningbo 315177, China; baojunli2006@163.com; 5 Central Office, Fenghua District Center for Disease Prevention and Control of Ningbo City, Ningbo 315500, China; hhxxttxx1981@163.com; 6 Department of Planned Immunization, Xinqi Street Community Health Service Center, Ningbo 315800, China; wangjingjing3326@126.com; 7 Department of STD and HIV/AIDS Control and Prevention, Ningbo Municipal Center for Disease Control and Prevention, Ningbo 315010, China

**Keywords:** repeated vaccination, seasonal influenza, elderly population, HAI titers

## Abstract

Background: Annual influenza vaccination is a WHO-recommended strategy for preventing seasonal influenza and its associated severe complications in older adults. Nevertheless, influenza vaccine effectiveness is often reduced in the elderly population and there remains an ongoing debate regarding whether repeated vaccination attenuates immune response. Methods: We conducted a prospective observational study to estimate the trivalent inactivated influenza vaccine-induced antibodies in older adults vaccinated for two consecutive years (2022–2023 and 2023–2024) and those with vaccines administered in a single season (2023–2024). Serum samples were collected concurrently with vaccination and at 30 and 90/180 days post-vaccination for hemagglutination inhibition (HAI) tests. Results: The participants administered two consecutive vaccinations had markedly higher pre-vaccination geometric mean titers (GMTs) and seroprotection rates for influenza A/H1N1 and A/H3N2. However, no intergroup differences were observed for H1N1, H3N2 or B/Victoria strains at 30, 90, or 180 days post-vaccination. At 30 days post-vaccination, participants with two consecutive influenza vaccinations showed significantly lower fold rises against the three strains and seroconversion rates (SCRs) for H1N1 and H3N2. The results of the subgroup analyses were largely consistent with the primary findings, with the exception of the A/H1N1 strain among individuals with pre-vaccination titers <1:10 at day 30. Conclusions: Immune responses vary by antigen type, and the influenza vaccine induces comparable serological response in the elderly, irrespective of their prior vaccination history.

## 1. Introduction

Seasonal influenza remains a significant public health burden, particularly among older adults who are at increased risks of severe illness, hospitalization, and mortality [1,2]. Annual vaccination is the most effective strategy for influenza prevention. In Zhejiang Province, the implementation of a voluntary free vaccination program for trivalent inactivated influenza vaccine (IIV3) has led to increasing numbers of elderly individuals receiving multiple vaccine doses across consecutive seasons. However, concerns persist regarding whether repeated influenza vaccination may attenuate immune responses in this population [3].

The impact of repeated vaccination on vaccine effectiveness remains debated. A meta-analysis of 41 studies [4] indicated that influenza vaccination in consecutive seasons may confer lower effectiveness than current-season-only vaccination. Our previous test-negative case–control study [5] found no significant difference in influenza risk between elderly individuals vaccinated in two consecutive seasons and those vaccinated only in the current season, indicating that repeated vaccination did not significantly compromise vaccine effectiveness. Nevertheless, most prior investigations [5,6,7,8] have focused on effectiveness outcomes using observational designs, leaving the immunological mechanisms underlying repeated vaccination incompletely understood.

Immunogenicity data are essential to complement effectiveness findings. Hemagglutination inhibition (HAI) antibody titers serve as a well-established correlate of protection against influenza. Yet, few studies have longitudinally tracked antibody dynamics throughout an entire influenza season in the same elderly individuals [9,10]. Moreover, age-related immune senescence may further complicate vaccine responsiveness, underscoring the need for population-specific immunogenicity data [11].

To address these gaps, we conducted a prospective observational study comparing IIV3-induced antibody responses in older adults who received two consecutive annual vaccinations (2022–2023 and 2023–2024) versus those vaccinated in a single season (2023–2024). By characterizing HAI titers at multiple time points post-vaccination, we aimed to evaluate the immunogenicity of current influenza vaccines and assess whether repeated administration modulates the magnitude or durability of antibody responses in the elderly.

## 2. Materials and Methods

### 2.1. Ethical Approval

The study was conducted in accordance with the Declaration of Helsinki and approved by the Ethics Review Committee of Ningbo Municipal Center for Disease Control and Prevention (protocol code 202208). Written informed consent was obtained from all participants prior to enrollment.

### 2.2. Participants Enrollment

Between August and September 2023, community-dwelling older adults were recruited from four districts of Ningbo City, China, namely Haishu, Jiangbei, Beilun and Fenghua. Sample size was calculated based on the primary objective of comparing geometric mean titers (GMTs) for the A/H1N1 strain at 30 days post-vaccination between the repeated- and single-vaccination groups. As shown in Equation (1), the calculation used the standard formula for two independent samples with log-transformed HAI titers, assuming a two-sided significance level of 0.05 and 80% statistical power. Based on an expected post-vaccination GMT of 24.9 for A/H1N1 in repeatedly vaccinated older adults [12], a mean difference of 1.0 between groups, and a standard deviation of 1.6 [13], the required sample size was 40 participants per group. After accounting for an anticipated 15% loss to follow-up, the target was increased to 47 participants per group. To ensure adequate power for multiple follow-up time points, the final enrollment target was conservatively set at 100 participants per group, yielding a total sample size of 200 participants.

(1) $$n=\frac{{\left(Z_{\propto /2}+Z_{\beta }\right)}^{2}*(\sigma_{1}^{2}+\sigma_{2}^{2})}{{\left(\mu_{1}+\mu_{2}\right)}^{2}}$$

The planned enrollment size was 50 participants per district, with purposive allocation according to their 2022–2023 influenza vaccination history. Specifically, 25 individuals per district had received the 2022–2023 seasonal influenza vaccine between August and December 2022, and the remaining 25 individuals per district had not been vaccinated during the 2022–2023 season and had no documented influenza infection.

Eligible participants were required to meet the following inclusion criteria: (1) aged 60 years or older; (2) scheduled to receive the standard-dose IIV3 for the 2023–2024 season at enrollment. Exclusion criteria comprised (1) immunocompromised conditions or severe chronic illnesses; (2) refusal to provide written informed consent or unwillingness to comply with study procedures.

Upon enrollment, all participants provided written informed consent and completed a structured questionnaire that collected demographic information, prior vaccination history, health status, and health-related behaviors. Supplementary data, including basic demographic information, clinical diagnoses and vaccination records, were retrieved from the Regional Health Information Platform (RHIP) of Ningbo City.

### 2.3. Sample Collection

Blood samples were collected from participants at baseline (concurrent with vaccination) and at follow-up time points. For participants in Haishu District, samples were obtained at 30 and 90 days post-vaccination, while for those in Beilun, Fenghua, and Jiangbei districts, samples were collected at 30 and 180 days post-vaccination. Sera were separated and stored at −80 °C until assayed.

### 2.4. Vaccines

All participants received the standard-dose trivalent split-inactivated influenza vaccine for the 2023–2024 season by intramuscular injection. The vaccine strains consisted of the Northern Hemisphere vaccine components shown in Table S1 and were provided by Shenzhen Sanofi Pasteur Biological Products Co., Ltd., Shenzhen, China.

### 2.5. Hemagglutination Inhibition Assay

The three influenza antigens used in this study were obtained from the National Institute for Biological Standards and Control (NIBSC): A/Victoria/4897/2022 (H1N1) pdm09 (Potency Number: 22/320), A/Darwin/9/2021 (H3N2) (Potency Number: 21/318), and B/Australia/1359417/2021 (Victoria lineage) (Potency Number: 21/316). According to the manufacturer’s instructions, serum samples were first treated with receptor-destroying enzyme (RDE; Denka Seiken Co., Ltd., Tokyo, Japan; Catalog No. 340122) and then heat-inactivated at 56 °C for 30 min prior to assay. Pre-treated sera were serially diluted two-fold in phosphate-buffered saline (PBS), beginning at a starting dilution of 1:10. Subsequently, 25 µL of antigen solution—adjusted to contain 4 hemagglutinating units (HAU)—was added to each well and incubated for 30 min at room temperature. Then, 25 µL of 1% guinea pig erythrocytes was added, followed by incubation for 1 h at room temperature. HAI titer was defined as the reciprocal of the highest serum dilution that completely inhibited hemagglutination. Samples with an HAI titer below 1:10 were assigned a nominal titer of 1:5.

### 2.6. Outcome Measures

The outcomes were measured using geometric mean titers (GMTs), seroconversion rate (SCR), seroprotection rate, and HAI fold change. SCR was defined as the proportion of participants exhibiting at least a four-fold increase in HAI titer to a minimum threshold of 1:40, measured 30 days after vaccination [10]. The seroprotection rate was defined as the proportion of participants achieving HAI titers of ≥1:40 following vaccination. HAI fold change was calculated as the ratio of post-vaccination to pre-vaccination HAI titers for each influenza strain.

### 2.7. Statistical Analyses

Given the skewed distribution of HAI antibody titers, all analyses were performed using log-transformed values to satisfy normality assumptions; results were subsequently back-transformed to the original scale for interpretation. GMTs, SCRs, and seroprotection rates were calculated for each vaccination group, along with their corresponding 95% confidence intervals (CIs). The HAI fold change was summarized as median and interquartile range (IQR).

To assess the independent effect of 2022–2023 influenza vaccination status on antibody responses to each vaccine component, multivariable linear regression models were constructed with log_10_-transformed GMTs or log_2_-transformed HAI fold change as dependent variables (Equation (2)). Covariates were selected a priori based on clinical relevance and potential confounding, including age, sex, prior influenza vaccination history from 2017–2018 to 2021–2022, multimorbidity status, drinking status, and exercise frequency. For binary outcomes (SCRs and seroprotection rates), multivariable logistic regression models with the same covariates were used to derive *p*-values (Equation (3)).

(2) Y = β_0_ + β_1_X_1_ + β_2_X_2_ + … + β_k_X_k_ + ε

(3) $$\mathrm{logit}(p)=\ln(\frac{p}{1-p})=\beta_{0}+\beta_{1}X_{1}+\beta_{2}X_{2}+\ldots +\beta_{k}X_{k}$$

Subgroup analyses were conducted using the same multivariable linear regression models with log_10_-transformed GMTs at 30 and 180 days post-vaccination as dependent variables to explore potential effect modification by each covariate (i.e., age, sex, multimorbidity status, influenza vaccination history, drinking status, and exercise frequency) (Table S2). Additionally, to control for confounding by prior influenza infection, participants were stratified by pre-vaccination HAI titer using a 1:10 cutoff, which represents the lower limit of detection and serves as a surrogate marker for pre-existing immunity, including subclinical infections [14].

All statistical tests were two-sided, and a *p*-value < 0.05 was considered statistically significant. Analyses were performed using Stata version 17.0 (StataCorp, College Station, TX, USA).

## 3. Results

### 3.1. Characteristics of Participants

In total, 210 volunteers were initially enrolled. After excluding nine participants who were lost to follow-up at 30 days post-vaccination, 201 participants were included in the final analysis from four districts of Ningbo City. The repeated-vaccination group comprised 110 individuals who received influenza vaccination in two consecutive seasons spanning 2022–2023 and 2023–2024, whereas the single-vaccination group consisted of 91 individuals who were un-vaccinated in 2022–2023 but received the trivalent inactivated influenza vaccine in 2023–2024. Baseline characteristics, including sex and alcohol consumption, were comparable between the two groups. Differences were observed in age, prior influenza vaccination history from 2017–2018 to 2021–2022, multimorbidity status, and exercise frequency. With respect to multimorbidity, the repeated-vaccination group had rates of diabetes at 40.00% compared to 24.18% in the single-vaccination group; hypertension was at 83.64% and 70.33%, and cardiovascular or cerebrovascular diseases were at 58.18% and 39.56%, respectively (Table 1).

### 3.2. Longitudinal Profiles of HAI Antibody Responses

Dynamic changes in immunogenicity were observed pre- and post-vaccination throughout the study period, and serological responses varied between the two groups at different sampling times (Table 2). A comparison of the HAI antibody trends for the three influenza vaccine strains in both groups reveals a rapid increase at 30 days when contrasted with the baseline. The GMTs in the single-vaccination group plateaued until 90 days and then decreased at 180 days, whereas the GMTs in the repeated-vaccination group began to decrease from 90 days, with the exception of A/H3N2. Immune responses exhibited variability according to antigen type, with pronounced variations observed in GMTs for H1N1 and H3N2, and more subtle changes observed for B/Victoria. The GMTs against the Victoria strain were consistently lower than those for H1N1 and H3N2.

**Figure 1:**
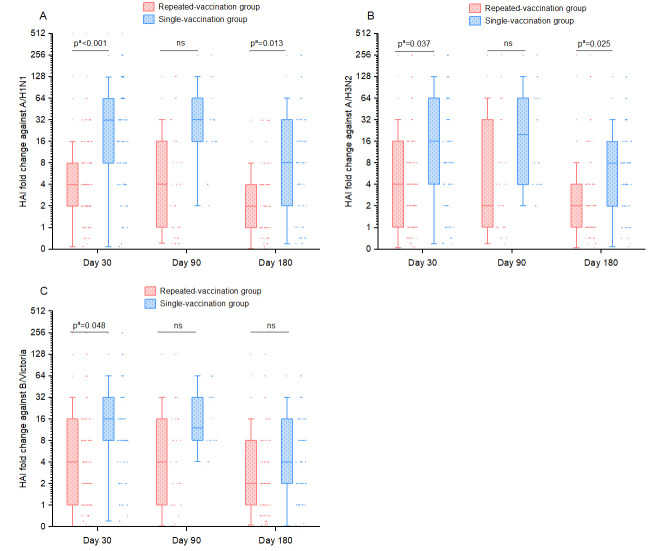
Comparison of median HAI fold changes against A/H1N1 (**A**), A/H3N2 (**B**) and B/Victoria (**C**) at 30, 90, and 180 days post-vaccination in single- versus repeated-vaccination groups among the elderly in Ningbo, 2023–2024. Each point represents the HAI fold change in the participants at different sampling times. The lines within the boxplots denote the median values, while the upper and lower boundaries correspond to the Q1 and Q3 percentiles, respectively. *p*^a^ values for HAI fold change were derived from multivariable linear regression models fitted to log2-transformed HAI fold change, with adjustment for age, sex, influenza vaccination history, multimorbidity status, drinking status, and exercise frequency. ns, not significant.

The baseline characteristics of the study participants exhibited significant imbalance between the groups. A range of demographic and health-related variables were entered into multivariable linear regression models to adjust for potential confounding factors, including age, sex, history of influenza vaccination, multimorbidity status, drinking status, and frequency of exercise. Following the adjustment for confounding variables, the findings indicated that the repeated-vaccination group exhibited significantly elevated GMTs for H1N1 and H3N2 in comparison to the single-vaccination group prior to vaccination (48.02 vs. 14.41 and 51.47 vs. 20.31, both *p* = 0.003) (Table 2). However, no intergroup differences were observed for the three vaccine strains at 30, 90, or 180 days post-vaccination. Following the administration of two consecutive influenza vaccinations at 30 days post-vaccination, participants exhibited a significantly reduced fold rise against all three influenza strains. At day 90, the fold changes were largely consistent between the two groups. By day 180, the fold changes against H1N1 and H3N2 were observed to be slightly elevated for the single-vaccination group (Figure 1).

### 3.3. Subgroup Analyses

Subgroup analyses were further performed based on these characteristics at 30 and 180 days post-vaccination (Table S2). The majority of subgroup outcomes exhibited congruence between the single- and repeated-vaccination groups. However, a salient observation was the elevated GMTs against H1N1 and H3N2 at 180 days post-vaccination in the repeated-vaccination group within the subgroup engaging in exercise at a frequency exceeding three times per week (232.90 vs. 155.04, *p* = 0.035; 213.57 vs. 136.68, *p* = 0.048).

**Figure 2:**
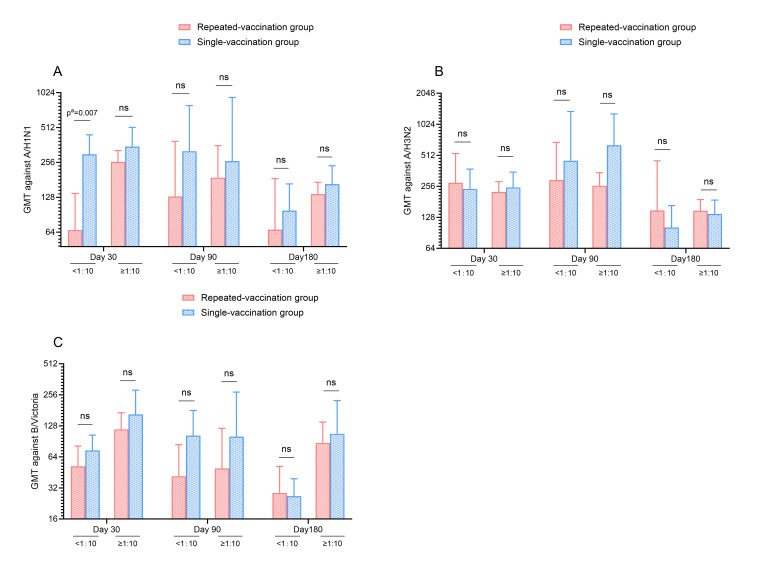
Comparison of GMTs against A/H1N1 (**A**), A/H3N2 (**B**) and B/Victoria (**C**) at 30, 90, and 180 days post-vaccination in single- versus repeated-vaccination groups with pre-vaccination HAI titers of <1:10 and ≥1:10 among the elderly in Ningbo, 2023–2024. *p*^a^ values for GMTs were derived from multivariable linear regression models fitted to log10-transformed GMTs, with adjustment for age, sex, influenza vaccination history, multimorbidity status, drinking status, and exercise frequency. ns, not significant.

To account for potential confounding by prior natural influenza infection, additional subgroup analyses stratifying participants within each vaccination group according to pre-vaccination HAI titers (<1:10 vs. ≥1:10) were performed. Following stratification, no statistically significant differences in GMTs against any of the three vaccine strains were observed between the single- and repeated-vaccination groups at any post-vaccination time point, with the sole exception of the A/H1N1 strain among participants with pre-vaccination titers <1:10 at day 30 (*p* = 0.007) (Figure 2).

### 3.4. Seroconversion and Seroprotection Rates

SCRs for H1N1 and H3N2 were found to be significantly lower in the repeated-vaccination group than in the single-vaccination group at 30 days post-vaccination (55.45% vs. 87.91%, *p* = 0.002 and 52.73% vs. 78.02%, *p* = 0.008). Given that SCRs were influenced by pre-vaccination HAI titers, we further compared the seroprotection rates. A higher baseline seroprotection rate was observed in the repeated-vaccination group compared to the single-vaccination group, with significant differences for H1N1 and H3N2 (63.64% vs. 26.37%, *p* = 0.001 and 67.27% vs. 39.56%, *p* = 0.012). Post-vaccination seroprotection rates against influenza H1N1 and H3N2 exceeded 90% in both groups and remained elevated for a period of six months. In contrast to the responses observed for H1N1 and H3N2, the Victoria strain demonstrated relatively transient immunogenicity. The seroprotection rates against the Victoria strain initially rose to over 80% following vaccination; however, a marked decline was observed by six months, which was shorter than the duration of H1N1 and H3N2. No significant intergroup differences in seroprotection rates were observed at any of the post-vaccination time points. It was evident that both groups exhibited robust and durable protection post-vaccination, although GMTs underwent a decline over time (Table 3).

## 4. Discussion

We examined trivalent inactivated influenza vaccine-induced antibody responses between a single vaccination and two consecutive vaccinations in the Chinese elderly population. Overall, our findings indicated that two consecutive influenza vaccinations did not significantly weaken antibody responses in the elderly population, consistent with our previous report [5]. Although a declining trend in HAI titers was observed in the repeated-vaccination group, this difference did not attain statistical significance after adjustment. The antigenic distance hypothesis, as a prevailing theory proposed by Smith et al. [15], posits that re-vaccination is most likely to interfere with serologic response when vaccine components remain unchanged or when the antigenic distance between strains in consecutive vaccines is minimal. According to the WHO recommendations for the 2023–2024 season, only the A/H1N1 vaccine strain was updated to reflect antigenic drift from prior strains. Specifically, the A/Victoria/2570/2019 strain included in the 2022–2023 season was replaced by A/Victoria/4897/2022 in 2023–2024, representing a shift from clade C to clade D. Nonetheless, no significant differences were observed in GMTs for all the three influenza vaccine strains between the single- and repeated-vaccination groups.

Furthermore, we observed a significant reduction in the fold rise in HAI titers among participants who were vaccinated for two consecutive years. Individuals with higher pre-vaccination HAI titers exhibited diminished fold rises in post-vaccination HAI titers, suggesting that pre-existing antibody levels might attenuate immune responses to subsequent vaccination with the same strain, a phenomenon previously proposed in the literature [16,17]. A cohort study [18] conducted in Hong Kong similarly reported that older adults who received vaccinations during either of the preceding two years demonstrated lower mean fold rises against all strains compared to those who did not. This result also accounted for the significantly lower SCRs against H1N1 and H3N2 observed in the repeated-vaccination group. It was reassuring to note that the seroprotection rates were not compromised by repeated vaccination. Notably, substantial baseline differences were observed between the repeated- and single-vaccination groups. The repeated-vaccination group had a higher proportion of participants aged ≥70 years and a greater prevalence of multimorbidity, including diabetes, hypertension, and cardiovascular or cerebrovascular diseases. Moreover, this group was characterized by a more frequent history of influenza vaccination in previous seasons, with 55.45% having received two or more doses from 2017–2018 to 2021–2022, compared with 7.69% in the single-vaccination group. These imbalances are of particular concern, as both advancing age and the presence of chronic conditions have been implicated in blunted humoral immune responses to influenza vaccination due to immunosenescence [19,20,21]. In addition, prior vaccination history may modulate current-season antibody responses through mechanisms such as immune imprinting or antigenic distance [15,22]. To mitigate the potential confounding effects of these factors, we employed multivariable regression models adjusting for age, sex, prior vaccination history, multimorbidity status, drinking status, and exercise frequency in all primary analyses. Furthermore, we conducted subgroup analyses stratified by these key covariates. Encouragingly, across the majority of subgroups, including those defined by age, multimorbidity and prior vaccination history, no significant differences in GMTs or seroprotection rates were observed between the two groups at any post-vaccination time point. The consistency of these findings across diverse strata, together with the multivariable adjustment, reinforces the robustness of our conclusion that repeated annual influenza vaccination does not meaningfully compromise serological protection in older adults, despite the presence of notable baseline differences.

It is crucial to acknowledge that the associations between vaccination history and immunogenicity are likely related to variations in natural influenza virus infections [23]. Regrettably, no instances of laboratory-confirmed influenza or related medication use were documented in the clinical records. To control for the potential confounding effect of prior natural infection, participants were stratified based on baseline HAI antibody titers using a threshold of 1:10, which serves as a surrogate marker for pre-existing immunity, including subclinical infections. The results of the subgroup analyses were largely consistent with the primary findings, revealing no significant differences in GMTs against the three vaccine strains between groups at any post-vaccination time point, with the exception of the A/H1N1 strain at day 30 among individuals with pre-vaccination titers <1:10, among whom repeated vaccine recipients exhibited a comparatively attenuated immune response. This attenuation likely reflects an enrichment of intrinsic low-responders exhibiting an “Insensitive to Influenza Vaccine” phenotype within the repeated-vaccination group, for whom a pre-vaccination titer <1:10 signifies a stable non-responsive state rather than immunological naïvety [24]. In addition, this selective manifestation in A/H1N1 likely stems from the antigenic stability of post-pandemic A/H1N1 strains relative to the rapidly evolving A/H3N2 [25,26]. Such stability permits repeated exposure to conserved epitopes, thereby revealing persistent non-responder phenotypes. Moreover, the pronounced immune imprinting observed with A/H1N1 [27], together with the stronger correlations between T-cell and antibody responses in influenza A compared to type B [28], may make intrinsic low-response patterns discernible only in A/H1N1 infections. In contrast, the greater antigenic variation and distinct immune dynamics of A/H3N2 and influenza B likely obscure such signatures.

Aging is associated with remodeling of the immune system, characterized by a reduction in naïve cells and an accumulation of dysfunctional memory cells [29], resulting in the attenuation of vaccine effectiveness in older age groups compared to younger populations. In this prospective observational cohort study conducted in a community-dwelling elderly population, we assessed the changes in HAI titers from the time of vaccination to six months post-vaccination to evaluate the immunogenicity and protective duration of IIV3 in the elderly. The trends in HAI antibodies for the three vaccine strains exhibited a marked increase post-vaccination, although the kinetics of antibody decline varied across strains. The GMTs in the single- and repeated-vaccination groups dropped at day 180 and day 90, respectively. Contrary to the prevailing assumption that booster immunization prolongs immune persistence, our findings suggest that repeated annual vaccination may be associated with a shorter duration of peak antibody responses. Notably, the GMTs for A/H3N2 remained elevated and even markedly increased in the single-vaccination group at day 90, which may be attributable to the A/H3N2 epidemic which occurred approximately three months subsequent to vaccination. This event provides a plausible explanation for the unusual phenomenon in our study, reflecting the combined effect of natural immunity and vaccination. Despite the observed reduction in antibody persistence among repeated vaccine recipients, seroprotection rates remained robust and significantly exceeded pre-vaccination levels, indicating that consecutive seasonal vaccination confers superior protection compared to no vaccination. Post-vaccination protection rates against H1N1 and H3N2 exceeded 90% in both groups and persisted for over six months. Although the protection rate for B/Victoria slightly decreased, it remained within the protective range throughout the influenza season. Consistent with most studies which indicate that HAI titers wane over time and are unlikely to sustain throughout the entire year in older adults [13], our findings underscore the need for continuous supplementation, particularly for B/Victoria.

Our previous large-scale test-negative design study [5], which included over 30,000 elderly individuals across four influenza seasons in the same region, found no significant increase in the risk of laboratory-confirmed influenza among those vaccinated in two consecutive seasons compared with those vaccinated only in a single season (adjusted OR 1.22, 95% CI 0.94–1.58). The consistency between the immunogenicity findings presented here and the real-world effectiveness evidence strengthens the conclusion that repeated annual influenza vaccination does not meaningfully compromise protection in older adults.

There are several study limitations. First, our study was constrained by a smaller sample size, and the use of a non-probabilistic opportunistic sampling may have introduced selection bias. In particular, the sample size at day 90 was imbalanced between the single- and repeated-vaccination groups, which may have reduced statistical power and affected the robustness of subgroup analyses at this time point. Similarly, some subgroup analyses were underpowered due to sample size attrition. Nevertheless, the main analyses at day 30 and day 180, as well as seroprotection rate outcomes, were based on larger and more balanced sample sizes, consistently supporting the core finding. In addition, despite the RHIP effectively reducing recall bias and minimizing the risk of misclassifying vaccine history and multimorbidity status, thereby enhancing the group comparability and estimation accuracy, systemic biases potentially affecting results cannot be entirely eliminated. Second, the vaccination data from two consecutive seasons cannot adequately capture the long-term effects of repeated administration on serologic response. Third, our evaluation was limited to the immune response to the vaccine antigens, without assessing their response to the circulating strains from that year, particularly antigenically drifted strains. In addition, HAI is just one correlate of protection against influenza; other immune parameters, such as neutralizing antibodies and T-cells, also contribute to host defense. These issues warrant further study with larger, more balanced cohorts incorporating a broader range of immunological indicators across multiple consecutive seasons.

## 5. Conclusions

In conclusion, our study suggests that the influenza vaccine elicits comparable immune response and confers sufficient protection in the elderly, irrespective of prior vaccination history. The current evidence continues to support the recommendation for annual influenza vaccination in the elderly population. Community health institutions should strengthen public awareness and actively promote voluntary influenza vaccine uptake to improve vaccination coverage among older adults.

## Figures and Tables

**Table 1:** Baseline characteristics of the study participants.

Variable	Overall	Repeated-Vaccination Group	Single-Vaccination Group	*χ^2^*	*p*
Total	201 (100.00)	110 (100.00)	91 (100.00)		
Age group (years), n (%)				34.21	<0.001
60–69	98 (48.76)	33 (30.00)	65 (71.43)		
70-	103 (51.24)	77 (70.00)	26 (28.57)		
Sex, n (%)				0.21	0.650
Male	83 (41.29)	47 (42.73)	36 (39.56)		
Female	118 (58.71)	63 (57.27)	55 (60.44)		
Multimorbidity					
Diabetes, n (%)	66 (32.84)	44 (40.00)	22 (24.18)	5.65	0.017
Hypertension, n (%)	156 (77.61)	92 (83.64)	64 (70.33)	5.08	0.024
Chronic respiratory diseases, n (%)	27 (13.43)	13(11.82)	14 (15.38)	0.54	0.460
Cardiovascular and cerebrovascular diseases, n (%)	100 (49.75)	64 (58.18)	36 (39.56)	6.91	0.009
Cancer, n (%)	19 (9.45)	10 (9.09)	9 (9.89)	0.04	0.847
Multimorbidity numbers, n (%)				11.16	0.011
0	25 (12.44)	12 (10.91)	13 (14.29)		
1	43 (21.39)	15 (13.64)	28 (30.77)		
2	46 (22.89)	31(28.18)	15 (16.48)		
3-	87 (43.28)	52 (47.27)	35 (38.46)		
Influenza vaccination history (2017–2018 to 2021–2022), n (%)				69.41	<0.001
Never	91 (45.27)	22 (20.00)	69 (75.82)		
Once	42 (20.90)	27 (24.55)	15 (16.48)		
Twice or more	68 (33.83)	61 (55.45)	7 (7.69)		
Frequency of drinking alcohol, n (%)				0.55	0.457
Never	56 (27.86)	33 (30.00)	23 (25.27)		
Once a week or more	145 (72.14)	77 (70.00)	68 (74.73)		
Frequency of exercise, n (%)				6.28	0.043
Never	50 (24.88)	35 (31.82)	15 (16.48)		
1–3 times a week	84 (41.79)	42 (38.18)	42 (46.15)		
More than 3 times a week	67 (33.33)	33 (30.00)	34 (37.36)		

**Table 2:** Comparison of pre- and post-vaccination GMTs in single- versus repeated-vaccination groups among the elderly in Ningbo, 2023–2024.

Virus Type	Time Point	Repeated-Vaccination Group		Single-Vaccination Group		*p* ^a^
		*n*	GMTs (95% CI)	*n*	GMTs (95% CI)	
A/H1N1	Day 0	110	48.02 (35.60–64.78)	91	14.41 (10.68–19.46)	0.003
	Day 30	110	204.57 (160.96–260.00)	91	324.91 (248.77–424.36)	0.323
	Day 90	31	127.94 (81.42–201.03)	14	320 (190.13–538.59)	0.416
	Day 180	73	126.19 (100.04–159.17)	65	133.47 (98.33–181.17)	0.969
A/H3N2	Day 0	110	51.47 (38.95–68.01)	91	20.31 (15.07–27.36)	0.003
	Day 30	110	233.52 (187.82–290.33)	91	246.99 (189.49–321.93)	0.444
	Day 90	31	267.59 (202.59–353.44)	14	579.66 (345.57–972.32)	0.690
	Day 180	73	148.30 (115.24–190.84)	65	123.87 (95.47–160.72)	0.139
B/Victoria	Day 0	110	19.02 (14.13–25.59)	91	7.32 (6.15–8.71)	0.053
	Day 30	110	82.56 (61.50–110.83)	91	89.68 (66.70–120.58)	0.461
	Day 90	31	44.73 (26.56–75.35)	14	102.47 (66.46–157.99)	0.947
	Day 180	73	57.93 (39.68–84.57)	65	40 (27.51–58.16)	0.934

*p^a^* values for GMTs were derived from multivariable linear regression models fitted to log10-transformed GMTs, with adjustment for age, sex, influenza vaccination history, multimorbidity status, drinking status, and exercise frequency.

**Table 3:** Comparison of seroconversion and seroprotection rates in single- versus repeated-vaccination groups among the elderly in Ningbo, 2023–2024.

Item	Time Point	Repeated-Vaccination Group		Single-Vaccination Group		*p* ^a^
Subtype		*n*	% (95% CI)	*n*	% (95% CI)	
**Seroconversion rates**						
A/H1N1	Day 30	110	55.45 (45.67–64.93)	91	87.91 (79.40–93.81)	0.002
A/H3N2	Day 30	110	52.73 (42.98–62.32)	91	78.02 (68.12–86.03)	0.008
B/Victoria	Day 30	110	52.73 (42.98–62.32)	91	79.12 (69.33–86.94)	0.153
**Seroprotection rates**						
A/H1N1	Day 0	110	63.64 (53.92–72.60)	91	26.37 (17.69–36.65)	0.001
	Day 30	110	94.55 (88.51–97.97)	91	96.70 (90.67–99.31)	0.511
	Day 90	31	93.55 (78.58–99.21)	14	100.00 (76.84–100.00)	N/A
	Day 180	73	95.89 (88.46–99.14)	65	95.38 (87.10–99.04)	0.916
A/H3N2	Day 0	110	67.27 (57.67–75.92)	91	39.56 (29.46–50.36)	0.012
	Day 30	110	97.27 (92.24–99.43)	91	94.51 (87.64–98.19)	0.656
	Day 90	31	100.00 (88.78–100.00)	14	100.00 (76.84–100.00)	N/A
	Day 180	73	95.89 (88.46–99.14)	65	93.85 (84.99–98.30)	0.744
B/Victoria	Day 0	110	35.45 (26.57–45.15)	91	9.89 (4.62–17.95)	0.110
	Day 30	110	80.00 (71.30–87.02)	91	83.52 (74.27–90.47)	0.964
	Day 90	31	67.74 (48.63–83.32)	14	100.00 (76.84–100.00)	N/A
	Day 180	73	65.75 (53.72–76.47)	65	60.00 (47.10–71.96)	0.847

*p*^a^ values were calculated using multivariable logistic regression models, with adjustment for age, sex, influenza vaccination history, multimorbidity status, drinking status, and exercise frequency. N/A, not available.

## Data Availability

The data presented in this study is available on request from the corresponding author.

## Supplementary Materials

The following supporting information can be downloaded at: https://www.mdpi.com/article/10.3390/vaccines14040338/s1, Figure S1: The study flow-chart.; Table S1: Viral antigens in the Northern Hemisphere’s trivalent inactivated influenza vaccine (IIV3) in the study.; Table S2: Comparison of post-vaccination GMTs between single- and repeated-vaccination groups across different elderly subgroups in Ningbo, 2023–2024.
