# Supplementary material for: Repeated Annual Influenza Vaccination in Older Adults Induces Comparable Seroprotection Despite Reduced Antibody Fold Rise: A 6-Month Prospective Cohort Study in China
Source: Vaccines (Basel). 2026 Apr 11;14(4):338. doi: 10.3390/vaccines14040338 (PMC13119716; doi:10.3390/vaccines14040338)
Supplement: Supplementary file 1 [file vaccines-14-00338-s001.zip › vaccines-4223087-supplementary.pdf]

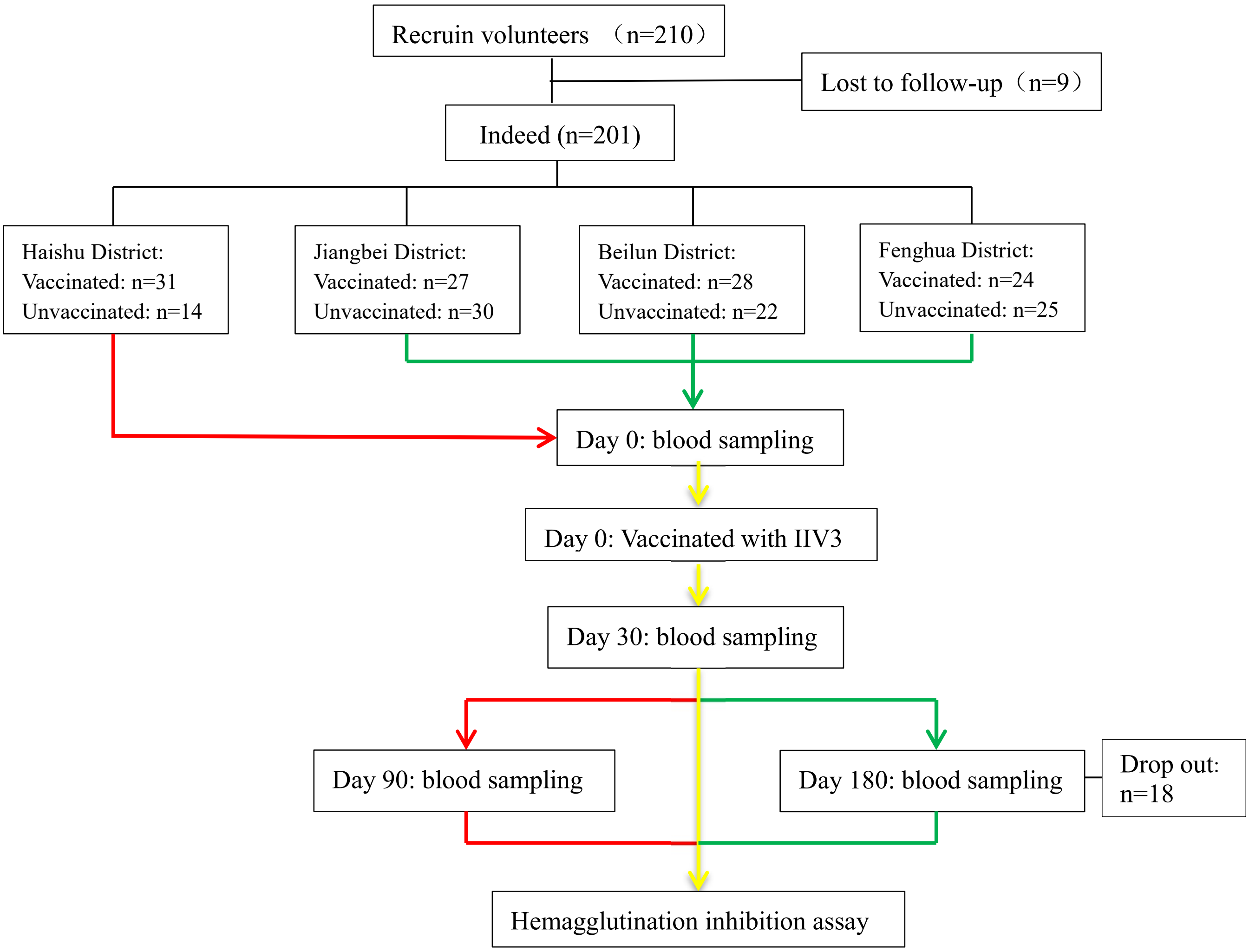

Figure S1. The study flow-chart. The red line represented the process for participants of Haishu District. The green line represented the process for participants of Jiangbei, Beilun and Fenhua Districts. The yellow line represented the process for the all participants.

Table S1. Viral antigens in the Northern Hemisphere's trivalent inactivated influenza vaccine (IIV3) in the study.

Season	Subtype	Vaccine strains
2017-18	A/H1N1	A/Michigan/45/2015 (H1N1) pdm09-like virus
	A/H3N2	A/Hong Kong/4801/2014 (H3N2) -like virus
	B/Victoria	B/Brisbane/60/2008 (B/Victoria lineage)-like virus
2018-19	A/H1N1	A/Michigan/45/2015 (H1N1) pdm09-like virus
	A/H3N2	A/Singapore/INFIMH-16-0019/2016 (H3N2)
	B/Victoria	B/Colorado/06/2017 (B/Victoria lineage)-like virus
2019-20	A/H1N1	A/Brisbane/02/2018 (H1N1) pdm09-like virus
	A/H3N2	A/Kansas/14/2017 (H3N2)-like virus
	B/Victoria	B/Colorado/06/2017 (B/Victoria lineage)-like virus
2020-21	A/H1N1	A/Guangdong-Maonan/SWL1536/2019(H1N1) pdm09-like virus
	A/H3N2	A/Hong Kong/2671/2019 (H3N2)-like virus
	B/Victoria	B/Washington/02/2019 (B/Victoria lineage)- like virus
2021-22	A/H1N1	A/Victoria/2570/2019 (H1N1) pdm09-like virus
	A/H3N2	A/Cambodia/e0826360/2020 (H3N2)-like virus
	B/Victoria	B/Washington/02/2019 (B/Victoria lineage)-like virus
2022-23	A/H1N1	A/Victoria/2570/2019 (H1N1) pdm09-like virus
	A/H3N2	A/Darwin/9/2021 (H3N2)-like virus
	B/Victoria	B/Austria/1359417/2021 (B/Victoria lineage)-like virus
2023-24	A/H1N1	A/Victoria/4897/2022 (H1N1) pdm09-like virus
	A/H3N2	A/Darwin/9/2021 (H3N2)-like virus
	B/Victoria	B/Austria/1359417/2021 (B/Victoria lineage)-like virus

Table S2. Comparison of post-vaccination GMTs between single and repeated vaccination groups across different elderly subgroups in Ningbo, 2023-24.

Subgroup	A/H1N1		p^a	A/H3N2		p^a	B/Victoria		p^a
	Repeated vaccination group	Single vaccination group		Repeated vaccination group	Single vaccination group		Repeated vaccination group	Single vaccination group	
**GMT at 30 days post-vaccination (95% CI, N=201)**									
Age group(years)									
60-69	243.54(142.07-417.48)	326.90(242.05-441.50)	0.435	294.21(193.28-447.85)	281.56(206.01-384.83)	0.998	64.84(39.90-105.39)	78.31(57.29-107.05)	0.483
70-	189.85(146.31-246.33)	320.00(176.93-578.77)	0.916	211.50(163.60-273.43)	178.01(106.75-296.82)	0.358	91.57(63.26-132.55)	125.87(62.28-254.38)	0.908
Sex									
Male	193.81(134.64-279.00)	279.65(179.96-434.56)	0.829	234.77(176.72-311.90)	201.59(135.08-300.84)	0.073	78.83(48.73-127.52)	91.54(56.92-147.22)	0.988
Female	212.99(153.61-295.31)	358.43(254.22-505.38)	0.283	232.58(168.46-321.11)	282.11(197.38-403.21)	0.870	85.46(58.46-124.94)	88.49(59.86-130.79)	0.656
Multimorbidity									
diabetes									
Yes	226.27(159.36-321.28)	351.72(179.51-689.15)	0.851	244.82(172.06-348.34)	256.67(123.58-533.07)	0.775	87.93(53.72-143.94)	145.57(69.28-305.88)	0.228
No	191.27(137.43-266.21)	316.80(236.93-423.60)	0.507	226.27(170.34-300.58)	243.98(185.46-320.96)	0.768	79.16(54.40-115.20)	76.85(56.14-105.20)	0.665
hypertension									
Yes	206.71(157.70-270.96)	309.77(221.43-433.35)	2.443	233.20(182.04-298.72)	246.75(174.89-348.14)	0.520	88.90(64.43-122.67)	100.43(71.13-141.80)	0.526
No	193.97(113.12-332.62)	363.83(231.95-570.68)	0.340	235.16(148.26-372.98)	247.55(166.56-367.91)	0.467	56.57(26.00-123.10)	68.58(37.80-124.41)	0.751
Chronic respiratory diseases									
Yes	198.04(88.18-444.74)	371.24(193.69-711.57)	0.849	417.76(239.80-727.81)	262.51(113.67-606.22)	0.074	99.02(31.69-309.37)	113.14(48.76-262.51)	0.120
No	205.47(159.19-265.20)	317.13(235.37-427.30)	0.682	216.00(170.90-273.01)	244.27(184.02-324.24)	0.366	80.57(59.33-109.43)	85.97(62.28-118.68)	0.375
Cardiovascular and cerebrovascular diseases									
Yes	209.75(150.11-293.10)	253.98(156.80-411.39)	0.811	238.87(173.66-328.56)	163.11(104.35-254.96)	0.318	94.11(62.23=142.32)	72.66(42.92-122.99)	0.992
No	197.58(139.30-280.24)	381.75(278.64-523.01)	0.249	226.27(169.82-301.50)	324.06(235.92-445.13)	0.709	68.81(45.29-104.55)	102.93(71.98-147.19)	0.620
Cancer									
Yes	183.79(77.13-437.96)	403.17(166.64-975.47)	0.174	171.48(80.55-365.08)	235.16(100.80-548.59)	0.235	121.26(27.05-543.62)	100.79(32.55-312.10)	0.381
No	206.78(160.42-266.54)	317.31(238.46-422.23)	0.299	240.84(191.25-303.29)	248.32(186.80-330.11)	0.552	79.45(58.97-107.04)	88.54(64.74-121.09)	0.599

Subgroup	A/H1N1		p[a]	A/H3N2		p[a]	B/Victoria		p[a]
	Repeated vaccination group	Single vaccination group		Repeated vaccination group	Single vaccination group		Repeated vaccination group	Single vaccination group	
Numbers of Multimorbidity									
0	213.57(102.07-446.89)	464.78(273.50-789.82)	0.939	226.27(123.13-415.82)	287.63(172.96-478.32)	0.956	84.76(43.68-164.45)	68.17(27.97-166.19)	0.645
1	201.59(89.10-456.07)	320.00(195.90-522.71)	0.494	278.58(161.25-481.26)	353.31(214.30-582.49)	0.892	69.64(31.38-154.58)	82.01(49.80-135.03)	0.966
2	191.34(128.37-285.20)	335.13(152.47-736.63)	0.251	213.97(134.65-340.02)	211.12(110.70-402.63)	0.947	74.81(43.48-128.73)	115.78(47.98-279.39)	0.809
3-	211.68(144.94-309.17)	284.15(176.81-456.64)	0.642	235.51(169.00-328.19)	187.47(116.00-302.96)	0.160	91.41(56.11-148.90)	95.61(57.72-158.36)	0.514
Influenza vaccination history (2017-18 to 2021-22)									
Never	351.72(187.10-661.20)	289.42(216.21-387.41)	0.567	320.00(170.58-600.30)	239.13(179.62-318.35)	0.341	85.20(40.89-177.53)	88.45(62.04-126.11)	0.519
1-2 times	235.16(180.04-307.15)	531.99(226.98-1246.86)	0.406	206.83(132.62-322.56)	242.51(98.23-598.71)	0.359	108.86(164.16-184.71)	83.78(40.88-171.72)	0.518
3- times	158.19(111.83-223.77)	353.31(106.92-1167.43)	0.310	219.94(167.84-288.21)	353.31(101.10-1234.66)	0.763	72.22(47.70-109.36)	118.88(37.19-379.98)	0.526
Frequency of drink alcohol									
Never	191.56(139.94-262.24)	323.28(233.94-446.73)	0.247	221.24(168.96-289.70)	248.01(179.62-342.46)	0.618	80.72(56.45-115.44)	84.18(59.36-119.39)	0.507
Once a week and more	238.47(170.30-333.94)	329.79(201.90-538.69)	0.777	264.88(181.12-387.38)	243.98(151.58-392.71)	0.137	87.01(57.44-150.11)	108.16(59.86-195.36)	0.657
Frequency of Exercise									
Never	139.29(98.12-197.72)	278.58(122.29-634.58)	0.295	144.92(109.11-192.46)	253.98(106.78-604.09)	0.573	80.00(42.72-149.81)	80.00(33.50-191.05)	0.682
1-3 times a week	185.62(118.79-290.05)	266.88(177.01-402.35)	0.467	249.83(164.01-380.55)	218.93(149.38-320.86)	0.998	76.14(46.55-124.54)	64.55(41.64-100.08)	0.493
More than 3 times a week	348.05(232.89-520.15)	443.42(301.14-652.92)	0.945	**355.44(248.25-508.91)**	**283.16(185.49-432.24)**	**0.030**	94.64(61.28-146.16)	141.58(90.51-221.47)	0.346
**GMT at 180 days post-vaccination (95% CI, N=138)**									
Age group									
60-69	130.71(83.14-205.50)	134.00(88.58-202.72)	0.790	201.59(124.99-325.12)	131.86(93.27-186.42)	0.078	33.64(18.70-60.50)	32.96(22.01-49.38)	0.405
70-	124.03(93.96-163.74)	132.44(84.73-207.02)	0.658	127.59(94.81-171.71)	109.63(73.54-163.44)	0.729	75.60(46.75-122.24)	58.38(26.15-130.35)	0.951
Sex									
Male	134.54(96.16-188.25)	117.94(71.81-193.70)	0.207	140.50(101.38-194.72)	108.53(69.52-169.43)	0.087	64.42(36.33-114.23)	47.24(23.75-93.95)	0.632
Female	120.03(86.15-167.25)	144.20(96.34-215.84)	0.481	154.68(105.58-226.63)	134.54(96.53-187.53)	0.608	53.32(31.55-90.09)	36.05(22.91-56.73)	0.772

Multimorbidity

Subgroup	A/H1N1		p^a	A/H3N2		p^a	B/Victoria		p^a
	Repeated vaccination group	Single vaccination group		Repeated vaccination group	Single vaccination group		Repeated vaccination group	Single vaccination group	
diabetes									
Yes	134.54(89.75-201.68)	134.54(63.11-286.81)	0.540	185.62(119.42-288.53)	95.14(45.73-197.91)	0.259	74.27(37.94-145.41)	56.57(21.39-149.61)	0.644
No	121.26(90.51-162.46)	133.12(94.80-186.93)	0.614	128.96(94.57-175.87)	135.02(103.58-176.00)	0.585	49.63(31.16-79.03)	35.72(23.92-53.34)	0.457
hypertension									
Yes	117.00(90.54-151.18)	129.57(92.61-181.27)	0.832	151.30(113.11-202.39)	120.17(86.95-166.06)	0.167	54.70(35.47-84.36)	45.12(28.67-71.03)	0.784
No	193.29(111.02-336.55)	143.41(70.13-293.27)	0.939	132.44(86.91-201.81)	133.32(83.29-213.40)	0.236	80.00(40.10-159.61)	29.88(14.73-60.59)	0.028
Chronic respiratory diseases									
Yes	160.00(69.94-366.05)	183.79(85.25-396.22)	0.386	237.76(114.94-491.84)	121.26(59.68-246.39)	0.517	44.16(6.83-285.50)	80.00(32.35-197.81)	0.529
No	123.05(96.04-157.67)	125.93(89.58-177.04)	0.608	141.05(107.62-184.87)	124.35(93.11-166.07)	0.184	59.62(40.33-88.14)	35.26(23.34-53.29)	0.513
Cardiovascular and cerebrovascular diseases									
Yes	116.76(87.14-156.45)	110.55(65.96-185.30)	0.913	141.05(97.16-204.78)	94.04(64.54-137.04)	0.179	53.96(31.86-91.38)	34.82(19.85-61.09)	0.414
No	141.98(95.26-211.60)	156.86(108.14-227.54)	0.476	160.00(116.75-219.27)	156.86(109.63-224.44)	0.093	64.52(37.03-112.42)	45.05(26.66-76.11)	0.208
Cancer									
Yes	126.99(42.48-379.66)	215.34(60.21-770.23)	N/A	285.09(56.36-1442.18)	160.00(76.32-335.43)	N/A	179.59(22.46-1435.77)	59.44(11.42-309.28)	N/A
No	126.12(98.84-160.92)	125.98(91.53-173.40)	0.564	139.87(108.91-179.62)	120.10(90.44-159.49)	0.326	52.35(35.75-76.65)	38.13(25.80-56.35)	0.763
Numbers of Multimorbidity									
0	226.27(113.20-452.31)	160.00(63.18-405.22)	0.299	134.54(73.85-245.11)	146.72(82.62-260.56)	0.960	80.00(35.25-181.55)	25.94(7.83-85.87)	0.693
1	86.40(30.33-246.16)	115.47(73.16-182.24)	0.447	172.81(76.21-391.85)	166.66(90.52-306.86)	0.363	46.66(15.18-143.47)	35.39(16.75-74.78)	0.087
2	135.66(96.15-191.41)	113.14(42.26-302.89)	0.410	126.99(80.98-199.15)	134.54(74.02-244.55)	0.969	82.68(41.58-164.43)	44.90(15.31-131.69)	0.128
3-	116.55(81.89-165.87)	148.55(88.40-249.61)	0.836	160.00(105.08-243.62)	95.14(62.65-144.48)	0.015	45.95(24.49-86.22)	46.41(25.57-84.21)	0.176
Influenza vaccination history (2017-18 to 2021-22)									
Never	160.00(84.65-302.43)	108.34(76.00-154.44)	0.373	209.50(108.31-405.24)	118.15(85.91-162.48)	0.119	68.58(32.00-146.96)	35.64(23.27-54.57)	0.336
1-2 times	133.53(100.03-178.26)	226.27(114.67-446.51)	0.723	108.14(81.46-143.55)	152.27(89.49-259.09)	0.116	49.39(22.89-106.59)	53.84(21.64-133.94)	0.180
3- times	106.02(73.68-152.55)	320.00(57.19-1790.40)	0.267	153.22(101.57-231.16)	100.79(7.26-1398.67)	0.580	59.07(33.25-104.94)	63.50(0.25-16091.16)	0.175

Subgroup	A/H1N1		p^a	A/H3N2		p^a	B/Victoria		p^a
	Repeated vaccination group	Single vaccination group		Repeated vaccination group	Single vaccination group		Repeated vaccination group	Single vaccination group	
Frequency of drink alcohol									
Never	114.66(84.16-156.21)	141.23(96.68-206.32)	0.660	155.79(112.15-216.41)	121.26(88.41-166.30)	0.096	51.53(32.02-82.93)	36.30(23.69-55.63)	0.934
Once a week and more	160.00(122.88-208.34)	110.55(70.09-174.38)	0.101	131.25(91.42-188.45)	133.00(83.18-212.66)	0.642	77.40(41.35-144.88)	55.28(23.47-130.19)	0.852
Frequency of Exercise									
Never	90.96(62.65-132.06)	122.56(50.79-295.75)	0.769	126.99(80.98-199.15)	160.00(69.23-369.78)	0.104	106.10(53.91-208.82)	49.51(19.29-127.07)	0.796
1-3 times a week	96.65(61.22-152.58)	124.09(78.07-197.24)	0.268	120.50(72.52-200.21)	103.15(75.27-141.35)	0.265	29.19(14.29-59.63)	18.23(11.40-29.16)	0.049
More than 3 times a week	232.90(170.66-317.85)	155.04(94.30-254.89)	0.035	213.57(146.73-310.87)	136.68(84.32-221.54)	0.048	54.96(31.84-94.85)	102.93(59.43-178.29)	0.193

p^a values for GMTs were derived from multivariable linear regression models fitted to log10-transformed titers, with adjustment for age, sex, influenza vaccination history, multimorbidity status, drinking status, and exercise frequency. In subgroup analyses stratified by a specific variable (e.g., age group), that variable was not included as a covariate. N/A, not available.
